# Integration of Care in Complex and Fragmented Service Systems: Experiences of Staff in Flexible Assertive Community Treatment Teams

**DOI:** 10.5334/ijic.6011

**Published:** 2022-05-25

**Authors:** Kristin Trane, Kristian Aasbrenn, Martin Rønningen, Sigrun Odden, Annika Lexén, Anne Landheim

**Affiliations:** 1 Inland hospital trust, NO; 2 Inland Norway University of Applied Sciences, NO; 3 Lund University, SE

**Keywords:** integration, integrated care, holistic, continuity, flexible assertive community treatment

## Abstract

**Introduction:**

To provide more integrated care, several countries have implemented the Flexible Assertive Community Treatment (FACT) model. However, this model does not guarantee full integration, especially in complex and fragmented service systems like in Norway. Hence, we investigated which barriers that might reduce the potential for integrated care in the Norwegian system, as described by staff in FACT teams, and how they adjust their way of working to increase the opportunities for integration.

**Methods:**

Online focus group interviews involving 35 staff members of five Norwegian FACT teams were conducted using a semi-structured interview guide. The material was analysed using thematic text analysis.

**Results:**

Six themes described the barriers to integrated care in the service system: fragmentation, different legislation and digital systems, challenges in collaboration, bureaucracy and limited opening hours. Three themes described adjustments in the teams’ way of working to enhance integration: working as the responsible co-ordinator, being a collaborator, and the only entry channel into the service system.

**Conclusion:**

The FACT team staff described several barriers to integration within the system. However, they made some adjustments in their way of working that might provide opportunities for integrated care within complex and fragmented service systems.

## Introduction

People with severe mental illness may have complex needs [1,2,3,4] and often experience mental health services as fragmented [5,6]. Fragmented services are a challenge in several countries [7,8,9,10,11,12,13,14,15,16,17], and better integration of care is needed [4,9,18,19,20,21,22]. Integration can be achieved in various ways [23,24,25,26,27], such as through multidisciplinary teams [18,23,28,29,30]. Flexible Assertive Community Treatment (FACT) is a multidisciplinary team model that is used in several countries [11,31,32,33,34,35,36,37] and aims to provide integrated care for people with severe mental illness [38]. However, such services do not guarantee the full integration of care [29,39]. Integration can be challenging [19,23,24,27,28,40,41], is context dependent [19,26,30,42,43] and might be especially difficult to achieve in complex health systems [41]. Hence, service systems can influence the potential for integrated care and how services work at improving opportunities for integration.

The concept of integrated care has various definitions within different health systems [27,28,30,44,45]. We consider this concept an umbrella term that includes holistic and continuous care [28]. Holistic care focuses on all aspects of people’s lives, including improving both health and perceived well-being [28]. Continuous care is defined as care that is connected and co-ordinated [17]. To provide integrated care, services need to take responsibility for treatment and be accessible [28]. The opportunities for integration may be improved by having common medical records [18,29,30,39,46], digital systems [19,24,28,39,47,48], flexibility [49] and clearly defined roles [18,25,50]. When a service, also called an actor, is unable to provide all treatments itself, co-ordination of services becomes important. This includes sharing information and managing transitions between services [28].

Knowledge about the other actors’ involvement [18], communication [28,47,51] and collaboration [9,23,40,52,53,54] are essential factors [18] for effective co-ordination. However, collaboration can be challenging [9,15,52,54] and can be hampered by the fragmentation of services [55], which contributes to less-integrated care. Different digital systems can hinder integration [15,25,30,42,52,56], especially given the importance of communication [25,28,52] in providing integrated care and because much communication is in writing [5] or through digital platforms [40]. Integration can also be hampered by bureaucracy [41], different levels of administration [9,52,57], poor co-ordination [18] and collaboration [52], and differences in regulations [26,36,52] and legislation [15,58]. Hence, complex and fragmented service systems can interfere with the opportunities for integrated care.

The FACT model was developed in the Netherlands [59] and has been implemented in countries such as Norway, Sweden [31,32] and Denmark [33,60]. In Norway, the health authorities support implementation of FACT teams if they are organized as a binding collaboration between the two levels of administration (primary and specialist care). This has resulted in about 70 FACT teams. The Norwegian formal mental health service system, henceforth referred to as the service system, includes many actors, such as inpatient and outpatient specialist health-care services, mental health and substance abuse services in primary care, the Norwegian Labour and Welfare Organization (NAV) and general practitioners (GPs) [16]. Norwegian FACT teams are largely an integral part of this system [61]. However, the fragmentation of services reduces their ability to provide all treatment themselves, especially because many services are regulated by different legislation. Hence, the FACT teams must work towards integrated care together with other actors in a system described as complex, fragmented [5,6,9,55,62,63,64,65] and bureaucratic [9,55], with poor collaboration [9,15,55,62,63,64,65] and co-ordination [5,66] and different medical records systems [65]. Some degree of parallel services has also been described [67] and should be avoided when integrated care is the goal [28]. Norwegian FACT teams are part of a system with barriers to integrated care, but to our knowledge, no studies have explored how complex and fragmented service systems influence the opportunities for integration within FACT teams and how teams adjust their way of working to increase the opportunities for integrated care. Greater knowledge about these aspects will contribute to further the understanding of factors that influence integration within such systems. In this study, we investigated the barriers staff in FACT teams described as reducing the opportunities for integrated care in the Norwegian system and how they adjust their way of working to increase the opportunities for integration.

## Methods

### Design

This study used a descriptive and partly inductive design with a qualitative approach [68]. To add to the existing knowledge [69,70], we conducted focus group interviews with the staff of five Norwegian FACT teams. Online interviews were conducted, and thematic text analysis was used to analyse the interviews [71].

### Setting and Sampling

This study is part of a larger study describing FACT teams in the Norwegian context and is financed by the Research Council of Norway. The FACT teams included in this study worked in different Norwegian regions, which differ in population density and geography. Using purposeful sampling [70], we included five teams for focus group interviews: three rural and two urban teams to cover different geographical contexts. Three of the teams were among the first FACT teams in Norway. The first was established in 2013, and the other two teams in 2018. All teams were organized within specialist health services and had a binding collaborative agreement with primary care. The case load and characteristics of the teams varied. Table 1 presents the characteristics of the teams.

**Table 1:** Characteristics of the Included FACT Teams.


TEAM	1	2	3	4	5

Organization	Specialist health service	Specialist health service	Specialist health service	Specialist health service	Specialist health service

Binding collaborative agreement between primary and specialist health care	×	×	×	×	×

Shared employer responsibility between primary and specialist health care		×	×	×	×

Included more than one local authority	×	×			×

Multidisciplinary teams	×	×	×	×	×

Number of patients	40	29	122	47	33

Number of team staff	7	9	11	8	10

Population in team’s region	24,000	23,500	58,500	30,400	18,600

Catchment area	4,327 km^2^	1,201 km^2^	7.5 km^2^	671 km^2^	1,289 km^2^

Type of region	Rural	Rural	Urban	Urban	Rural


Thirty-five staff members of five different FACT teams were recruited to participate in the five focus group interviews. To ensure diversity of experiences, we invited all staff members of the teams, but not all of them could participate. The participants represented all the different roles and specialist functions within the teams (Table 2). Some had more than one role, such as being a case manager and providing a specialist function. In such cases, the participant’s specialist function was recorded, and the actual number of case managers is higher than shown in Table 2. The case managers’ professional background varied, but many were social workers, medical nurses or social educators.

**Table 2:** Participants in the Focus Groups.


FOCUS GROUP INTERVIEWS	1	2	3	4	5	TOTAL

Team leaders	1	1	1	1	1	5

Psychiatrists		1	1	1		3

Case managers	2	3	1		4	10

Peer specialists		1		1	1	3

Employment specialists	1	1	1	1		4

Psychologists	1	1	1			3

Substance abuse specialists			1	1	1	3

Others		1		2	1	4

Total number of participants	5	9	6	7	8	35


### Data Collection

The interviews were conducted in January and February 2021. A semi-structured interview guide was used. This guide focused on integration of care, collaboration, and barriers to and drivers of integrated care. The interview guide was pilot tested [70] in collaboration with a FACT team leader from a team not included in the study, and this led to some adjustments. Because of the COVID-19 pandemic, the interviews were conducted online. In four of the interviews, some participants sat together, and the others participated individually from a separate computer. In one interview, all participants sat together, and the researchers participated digitally. Two authors conducted the interviews, and the first author took the leading role. The focus group interviews lasted for 60–80 minutes and were digitally recorded.

### Data Analysis

The interviews were transcribed verbatim. After completion, all interviews were analysed using thematic text analysis, as described by Braun and Clarke [71]. Data were analysed across the interviews using a partly inductive approach [71]. We first listened to all interviews and read through the transcripts looking for patterns. The data were then coded, and the codes were given names related to their content. In the next step, we grouped the initial codes into themes and gave them descriptive names. We next read through the transcripts once more to check how well the themes and data were connected. We then moved some themes and changed the names of others. We also examined the data for exceptions [68]. We discussed both themes and sub-themes and made further changes. We then wrote a summary of each sub-theme. Throughout the process of writing the article, we returned to the data, made some minor changes to themes and sub-themes, and read through the transcripts several times.

### Ethical Considerations

The study was approved by the Data Protection Officer for South-Eastern Norway (ID 104187). Participants provided written informed consent to participate. The consent letter contained information about the purpose of the study, safekeeping of the data and how participant confidentiality and anonymity would be ensured.

## Results

Six themes were found to describe the barriers to integrated care in the service system: fragmentation, different legislation between services, different digital systems between services, challenges in collaboration, bureaucracy and limited opening hours. Three themes described how the teams adjusted their way of working to enhance the opportunities for integration: focus on working as the responsible co-ordinator, being a collaborator and being the only entry channel into the service system.

### Barriers to Integrated Care in the Service System

#### Fragmentation

Different service levels and the large number of different services were mentioned by several participants as obstacles to integrated care. One challenge was informing all actors about the FACT team. Participants reported that the large number of services required excessive time in meetings, which created difficulties when performing their role as a co-ordinator and reduced the time they could spend on direct contact with patients. A substance abuse specialist in a rural team stated:

“To get things really well integrated takes quite a bit of time and prioritizing.”

The large number of actors meant that many services shared responsibility and tasks. Although the division of tasks and responsibilities between the FACT teams and some of their partners worked well, several team members noted ambiguities in this division. Poor knowledge about the FACT team was noted as increasing this lack of clarity. One asked:

“Where do we stop and where does someone else’s work begin?”

#### Different legislation between services

Participants from all FACT teams highlighted that different legislation hampered the opportunities for integrated care. They felt that this made it impossible to provide services for the NAV, for inpatient care, for GPs and for emergency services, and resulted in different medical records and other digital systems within the various services. Different legislation was also described as making it difficult to share information because of privacy issues. This was described as hampering co-operation and information exchange and opportunities for integrated care.

#### Different digital systems between services

Participants from all teams perceived that the different digital systems, including different medical records systems, act as a barrier to integrated care. This was described as challenging and that it sometimes leads to errors in medication. Several team members said that they could not share crisis plans with emergency services, and one stated that the only way they could do so would be to deliver a “wheelbarrow full of crisis plans”. Another participant said that having the same digital systems would “really help co-ordinate between the services”. A team leader in a rural region stated:

“Primary mental health and substance abuse have different medical record systems; the emergency services have one system and GPs have another one. Poor flow of information makes it difficult to have good services.”

#### Challenges in collaboration

Participants in all teams described being dependent on collaboration with other actors to provide integrated care. One said that, without collaboration, “the team gets stuck”. NAV was highlighted as important for providing holistic care. A psychologist in an urban region explained:

“NAV is especially important for our target group. These people have lots of financial problems and own next to nothing. They’re completely dependent on NAV processing their application for benefits so that they can pay their bills and won’t get thrown out of their flat.”

In all teams, participants described challenges in collaboration with some actors, especially NAV. Some mentioned that it was difficult to contact NAV and that privacy issues made co-ordinating with them challenging. One called NAV a “closed partner”. These issues were described as hindering integration. Some also described collaboration challenges with GPs, emergency services and inpatient services. In one team, collaboration challenges were emphasized as the main barrier to integrated care. An employment specialist explained:

“We try to work in the patients’ best interests by collaborating, but sometimes we find closed doors. It’s difficult to position ourselves and we’re not given a direct phone line.”

#### Bureaucracy

Bureaucracy, especially requirements for documentation, was described by many participants as a barrier to integration. When asked to name a factor that could improve integration, a team leader in a rural region replied:

“Then I’d do something about the registration and documentation tyranny that takes up valuable time, that takes us away from patients and prevents us from being pragmatic and flexible in the way we relate to them.”

One participant said the documentation requirements were increasing, and several stated that these requirements consume too much time. A peer specialist in a rural team said:

“This takes time that could have been spent on useful things for patients.”

#### Limited opening hours

Several team staff were concerned about being accessible to patients, and some found it challenging that they were available only during office hours. One described this as something out of their control to change and explained:

“Only working during office hours is a limitation since working to include people as part of the community often happens after four o’clock”.

In one team, the staff said that “they make sure that they give patients their telephone number” because they were also available by phone in the afternoons and evenings.

### Adjustments of the Team’s way of Working to Increase the Opportunities for Integration

#### Focus on being the responsible co-ordinator

Participants from all teams reported that they considered the team as the service most responsible for ensuring holistic treatment. One called this “having a firm grip on the cases”. Another said, “We can never give up our overall responsibility for treatment.”

The team members reported that because they are unable to provide all treatment and care themselves, they need to both co-ordinate services provided by the team and by services outside the teams. One described this as a “co-ordinator function with responsibility for continuity of care to ensure that things get done”.

The participants noted that to co-ordinate services, they need to have a good overview of the treatment that they and other services provided, as well as the patient’s needs. Most team members said that they generally have such knowledge, despite the challenges. They often made comments such as, “We have a pretty good idea, or we mostly know what we need to know”. Others also commented that “it’s challenging.” The daily meetings were described as helpful in providing an overview. One also described using treatment plans to keep track of the services they and other actors provided, and said:

“We use the treatment plan to clarify future treatment with other services”.

#### Focus on being a collaborator

All participants noted that providing integrated care in the Norwegian service system required collaboration with several other actors. Although some said that they could work harder to achieve collaboration, many described working consciously to establish good relationships by initiating regular meetings, being flexible and accessible, and contacting other services often. A team leader in a rural region said, “We have reached out a hand in many directions”. Many participants described this as time-consuming. A team leader in a rural region explained:

“We’re working to get a place in the system. There hasn’t been any FACT team here before, so we needed to establish our intended role”.

#### Focus on being the only entry channel into the service system

To provide integrated care, several participants described that they tried to be the only entry channel into the service system. This included having a holistic focus, being accessible, providing several services themselves and being the patients’ guide through the system. They noted that they must work hard to create continuity of care. Many said they attempt to be the patients’ only entry channel into the service system to avoid patients having to deal with different services. They stated that patients should be able to contact the FACT team directly and that they would “ensure connection to other services” and “accompany patients on their path through the various services”. One team leader in a rural region explained:

“There’s a common thread that runs through their treatment anyway. They can always contact us, whether they have questions about doctors, hospitals, mental health centres, NAV or anybody.”

Several team members described how they “guide patients through the system” before, during and after inpatient stays. One case manager in a rural region said:

“If he’s admitted to specialist treatment, we’re there. If he’s in an inpatient facility, we’re there. We go with them to primary health care, to specialist treatment, when they’re admitted and when they come out again.”

Many described having a holistic focus on the patients’ needs and attempting to adapt support to the individual patient’s needs. They described attempting to address all elements of their patient’s life that may affect their health, such as housing, finances, networks, work and physical health. A team leader in an urban region said:

“We focus on providing help that’s actually seen as help, and when we meet new patients who live in a rubbish dump, have no teeth, have very poor physical health, have no money, well, that’s where we have to start.”

Some said that their team provides much treatment and care. Descriptions such as, “We have many services within the team”, were common. The team members described how their work involves a variety of elements, such as counselling, medication, environmental therapy, exposure therapy, psychoeducation, following up physical health status and accompanying patients to other treatments. They also performed practical work if needed or when they felt that it would help in building a relationship with the patient.

## Discussion

The FACT team staff members described several aspects of the service system as constraining their ability to provide integrated care. However, they made some adjustments in their way of working to enhance the opportunities for integration.

Our study findings indicate that the ability of FACT teams to provide integrated care are hampered by different services and levels of administration, differences in legislation and digital systems. These findings are consistent with other observations about the barriers to integrated care. A study that interviewed partners of FACT team members also found these factors to hamper collaboration [61]. Other studies have found that different laws [58], levels of administration [63] and digital systems [15,52] can be barriers to collaboration. Much communication is in writing, and the use of incompatible information exchange systems can have negative consequences for patients [47,72]. The opportunities for integration were also described as being hindered by bureaucracy, especially because documentation consumes time. Manoeuvring between different services in fragmented systems has been described as consuming time that could have been spent on direct patient-oriented work [55]. Together with the barriers to both collaboration and integration, this limits the opportunities for integrated care.

The participants noted that co-ordinating services is challenging, partly because of the diverse services the teams had to co-ordinate and have an overview of, as well the collaboration challenges. The wish to collaborate does not necessarily mean that collaboration will occur [73]. One reason for challenges in collaboration is a vague understanding of roles [1]. The FACT team staff described this partial lack of understanding: a finding that was also reported in a study that interviewed partners of FACT teams [61]. The lack of clarity may make it more difficult to provide integrated care [15,39,62] and indicates the need for role clarification. The teams’ dependence on other services was described as another challenge to the provision of holistic care, for example, patients having to wait for basic needs to be met. NAV was highlighted as a service they were particularly dependent on but was described as difficult to co-operate with. In Norway, NAV makes decisions about housing and financial support [16], which makes the FACT teams dependent on them when providing holistic care. Inclusion of NAV in FACT teams might enhance integration by allowing teams to address financial and housing issues. However, this is difficult within the Norwegian service system because of differences in legislation.

According to the FACT model, teams are supposed to have 24/7 accessibility [38]. The availability of the FACT teams only during office hours was described as limiting the opportunities for integration, and other services have called for extended opening hours [61]. One of the FACT teams noted that they were accessible to patients after office hours, which indicates that they were trying to overcome this barrier. How this accessibility was organized did not emerge from our study but should be further investigated. However, focusing on all aspects of people’s lives also includes the life they live after office hours, and accessibility is important when aiming to provide integrated care.

The FACT teams described their adjustments in their way of working to strengthen the opportunities for integration. To contribute to continuity of care, the FACT team staff had expanded the role of the responsible co-ordinator. According to the FACT model, teams take responsibility for patients, provide most services themselves and co-ordinate the care and treatment they provide [38]. However, because of the number of services, Norwegian FACT teams are unable to provide all care themselves and they must co-ordinate several services outside the team. This was described as challenging, which implies that providing as much treatment and care as possible within the FACT team may increase the opportunities for integration. This may reduce the number of services the FACT team needs to have an overview of, provide more time for direct patient contact and enhance the opportunities for integration.

FACT teams are supposed to have daily meetings in which the multidisciplinary team co-ordinates care and support, and all patients are supposed to have treatment plans [38]. The participants described this as contributing to a better overview of both the services provided by the team and other services. Danish FACT teams use treatment plans to create a common vision with inpatient care [60]. Having a common vision may promote collaboration [74,75] and integration of care [24,25,28,75], and common treatment plans may promote collaboration [49]. This suggests that the opportunities for integration may be enhanced if FACT teams invite patients and other services to participate in developing treatment plans. This might also facilitate collaboration and contribute to clarification of roles and tasks, and highlights the importance of the responsible co-ordinator when complexity and fragmentation characterize the service system.

Communication [28,47,51] and collaboration [9,23,40,52,53,54], are crucial to the co-ordination of services, and the FACT teams described the need for collaboration with other services. They seem to have taken a role as a collaborating actor in the service system by creating relationships with other services. Swedish [76] and Danish [60] FACT teams collaborate with other actors, and Danish FACT teams describe consciously working to build relationships with other services [60]. Norwegian FACT teams have been described by other service providers as working to promote collaboration and improving collaboration in the service system [61]. However, a study that interviewed patients from the same study as ours noted the potential for improvement in their collaboration with other services [77]. Our findings indicate that collaboration is essential for FACT teams in complex and fragmented systems.

The FACT team staff described efforts to create holistic and continuous care by focusing on being the only entry channel into the service system, guiding the patients through the different services, being accessible and providing as much treatment and care as possible within the team. This way of working may promote the opportunities for integration. The previously mentioned study that interviewed patients from the same FACT teams as our described that being treated as a whole person and finding help with all their challenges from the same team made them feel safer, improved their daily life situation and made it easier to relate to the service system [77]. This indicates that FACT teams can contribute to better integrated care even in complex and fragmented service systems.

### Strengths and Limitations

Whether the FACT teams provide integrated care can be answered only by their patients. Nevertheless, the process of integration can be studied from the perspective of service providers [27,28], as in our study. We used focus group interviews to increase understanding of how services such as FACT teams work towards their goal of integrated care in a system characterized by its complexity and fragmentation, and which barriers they have experienced within this system. We do not know how the teams actually work but only how they describe their work. To ensure a common understanding, we asked the team staff members to share their understanding of the concept of integrated care. They described a view that matches both the definitions and common values regarding integrated care [45]. The same interview guide was used in all interviews. Participants were asked to respond openly when describing the barriers to, and drivers of, integrated care. Barriers within the Norwegian service system were emphasized. Descriptions from rural and urban teams included more issues in common than anticipated prior to the interviews. However, challenges specific to rural regions were not specifically addressed and should be studied further.

A limitation of our study is that the interviews were conducted online. However, our interviews provided rich data and the participants interacted well, possibly because they were used to virtual meetings. All specialist roles in the FACT teams were represented, which increases the transferability of the study findings [78]. We have used quotations from participants who play different specialist roles in the teams and have stated their role in the teams and whether they represented an urban or a rural team.

Focus group interviews imply several ethical issues [70,79], such as participants having relationships that continue after the interviews. This might have affected what was said in the interviews. Differences in how much each participant spoke were observed, which might have reduced the credibility of this study [78]. However, those who talked less were asked specific questions several times. The participants might have felt it difficult to decline participation in this study because the interviews were performed as part of their job. However, not all members of the included FACT teams participated. After analysis, the findings were presented to nine staff members, who in total represented all the included FACT teams. They were asked if the findings were recognizable, and all said that the findings were “very recognizable”. This increases the study’s credibility [78,80].

Our study is relevant to settings in countries other than Norway and especially those with complex and fragmented service systems. It is also relevant to similar health services that cannot provide all treatment themselves and for health authorities, politicians and leaders who make decisions regarding the organization of services within such health systems.

## Conclusion

Providing integrated care in FACT teams part of a complex and fragmented service system is challenging. Several factors hamper integration such as the involvement of many services, different levels of administration, differences in legislation and digital systems, bureaucracy, challenges in collaboration and not being accessible outside of office hours. These structural barriers consume time that could been used for direct patient-oriented work and make it difficult for the FACT teams to maintain an overview. FACT teams in such service systems seem to be working in a “structural headwind”. Teams or similar services that are part of such systems may be unable to provide truly integrated care unless some of the structural barriers are dealt with.

The FACT teams actively made adjustments to increase the opportunities for integration within a complex and fragmented system. One adjustment was identified as the theme of focusing on being the responsible co-ordinator, which seems to increase in importance with the number of services and other barriers to integration that are involved. Finding ways to ensure a good overview seems to be essential and implies that FACT teams require an extra focus on prioritizing the daily meetings. Integration may also be enhanced by including collaborating actors in developing treatment plans. However, this also implies that the opportunities for integration of care seem to increase if the FACT teams provide as much treatment and care as possible themselves. The more services that are provided outside the teams, the greater the collaboration needed becomes, which can be both time-consuming and challenging. When the systems are characterized by complexity and fragmentation, focusing on being a collaborator seems essential. This implies the need for FACT teams or similar services in such systems to work consciously in ways that enhance collaboration. To provide holistic and continuous care in such systems, being the only entry channel into the service system also seems essential. Succeeding in this might reduce patients’ experiences of fragmentation and increase the opportunities for integration.
